# TRPA1 as a drug target—promise and challenges

**DOI:** 10.1007/s00210-015-1088-3

**Published:** 2015-02-03

**Authors:** Jun Chen, David H. Hackos

**Affiliations:** 1 Department of Biochemical and Cellular Pharmacology, Genentech, South San Francisco, CA 94080 USA; 2 Department of Neuroscience, Genentech, South San Francisco, CA 94080 USA

**Keywords:** TRPA1, Drug target, Promise, Challenges

## Abstract

The transient receptor potential ankyrin 1 (TRPA1) channel is a nonselective cation channel belonging to the superfamily of transient receptor potential (TRP) channels. It is predominantly expressed in sensory neurons and serves as an irritant sensor for a plethora of electrophilic compounds. Recent studies suggest that TRPA1 is involved in pain, itch, and respiratory diseases, and TRPA1 antagonists have been actively pursued as therapeutic agents. Here, we review the recent progress, unsettled issues, and challenges in TRPA1 research and drug discovery.

## Introduction

**Fig. 1:**
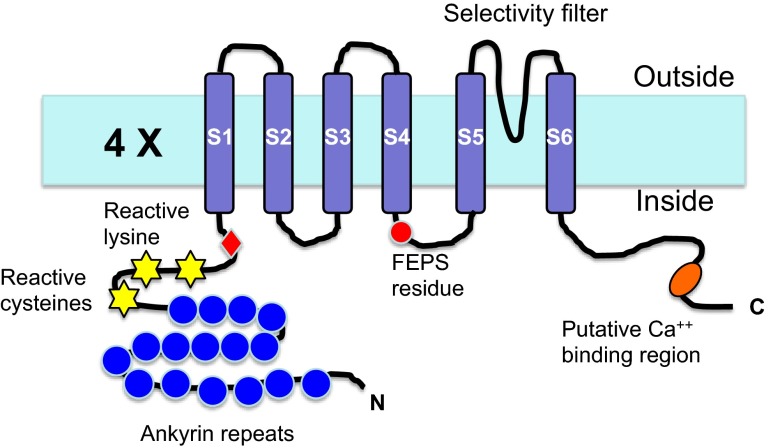
Structure of the TRPA1 channel. The TRPA1 channel shares the overall architecture of voltage-gated ion channels. It is a homotetramer with each subunit containing six transmembrane helices and intracellular N- and C-termini (as shown). The transmembrane helices are labeled S1–S6 with S1–S4 representing the ancestral voltage-sensing domain (*VSD*) and S5–S6 forming the central pore and selectivity filter. The reactive lysine and cysteine residues are shown within the N-terminal domain, along with the N-terminal ankyrin repeats. Please note N855S, the residue mutated in familial episodic pain syndrome (*FEPS*), is shown on the intracellular end of S4 based on recent electron cryo-microscopy structure of TRPV1 (Liao et al. 2013) and comparison between TRPA1 and TRPV1 hydropathy plots, and the putative Ca^++^ binding region is shown within the C-terminus

Transient receptor potential ankyrin 1 (TRPA1) is one of the 28 members of the transient receptor potential (TRP) channel family and the sole member of the TRPA subfamily in mammals. Like all TRP channels, TRPA1 possess a tetrameric structure with a single pore present at the central axis. Each subunit contains six transmembrane alpha helices (labeled S1–S6) and intracellular N-terminal and C-terminal domains (see Fig. 1). The pore-forming selectivity filter is positioned between the S5 and S6 transmembrane helices. TRPA1 is unusual among mammalian TRP channels in having a very long ankyrin repeat within the N-terminal domain (14–18 ankyrin repeats depending on species). TRPV and TRPC channels also have N-terminal ankyrin repeats, although they are much shorter (three to six repeats). TRPA1 is permeable to both monovalent and divalent cations, and therefore, TRPA1 is capable of depolarizing the membrane and initiating Ca^2+^ signaling in the cells it is expressed.

## Expression pattern

TRPA1 is highly expressed in small- and medium-sized peptidergic primary afferent somatosensory neurons present in sensory ganglia-containing nociceptors—the dorsal root ganglia (DRGs), the trigeminal ganglia (TGs), and the nodose ganglia (NGs) (Nagata et al. 2005). Depending on different reports, the fraction of DRG neurons expressing TRPA1 varies from 3.6 to 56.5 % (Story et al. 2003; Nagata et al. 2005; Bautista et al. 2006; Kwan et al. 2006; Niforatos et al. 2007), with the most commonly reported values being around 30 %. The capsaicin receptor TRPV1 appears to be co-expressed in most if not all TRPA1-expressing DRG neurons (Bautista et al. 2006; Anand et al. 2008). This finding is further supported by the observation that mustard oil-induced nocifensive behavior is eliminated in mice where the central terminals of TRPV1-expressing DRG neurons have been ablated by intrathecal injection of capsaicin (Shields et al. 2010). In addition to TRPV1, TRPA1-expressing nociceptors also express calcitonin gene-related peptide (CGRP), substance P, and the bradykinin receptor, which are key mediators/transmitters in nociceptive signaling (Jordt et al. 2004; Obata et al. 2005; Bautista et al. 2006).

TRPA1 expression outside of nociceptive neurons has been reported by many groups, though the results do not always have the same level of consistency as seen in DRG and TG neurons. Nonetheless, expression in such cells represents potential locations where selective TRPA1 antagonists might have on-target effects outside of pain. Hair cells in the inner ear were reported to express TRPA1 at both the RNA and protein level as determined by in situ hybridization and immunohistochemistry, respectively (Corey et al. 2004; Nagata et al. 2005). As such, TRPA1 was proposed to be a component of the hair cell tip-link mechanotransducer channel necessary for auditory transduction. However, further experiments with TRPA1 knockout (KO) mice demonstrated that TRPA1 appears not to contribute to hair cell transduction or auditory function in vivo (Bautista et al. 2006; Kwan et al. 2006). Sympathetic neurons such as those of the superior cervical ganglion (SCG) have been reported to express TRPA1 (Smith et al. 2004), though other groups have failed to detect significant levels of TRPA1 RNA in the SCG (Nagata et al. 2005; Munns et al. 2007). Myenteric neurons and enterochromaffin cells (as well as some nonneuronal epithelial cells) in the small and large intestine have also been proposed to express TRPA1 based on immunohistochemistry and RT-PCR (Anand et al. 2008; Nozawa et al. 2009; Poole et al. 2011; Kono et al. 2013). Furthermore, treatment of enterochromaffin cells with TRPA1 agonists induces serotonin release, and treatment of the isolated guinea pig ileum with allyl isothiocyanate (AITC) induces 5-HT3-receptor-mediated gastrointestinal smooth muscle contractions. TRPA1 agonists have been further shown to delay gastric emptying in rats through this pathway (Doihara et al. 2009). However, it is not clear whether TRPA1 antagonists would have deleterious effects on gut motility.

Nonneuronal expression of TRPA1 has been reported by many groups. In the lung, besides its expression in innervating sensory fibers, TRPA1 has been detected in several nonneuronal cell types including lung fibroblasts, alveolar epithelial cells, and lung smooth muscle cells in both human and mouse (Mukhopadhyay et al. 2011; Nassini et al. 2012), though only very low levels of TRPA1 RNA in the mouse and human lung were detected in other reports (Jaquemar et al. 1999; Story et al. 2003; Jang et al. 2012) (author’s unpublished data). In skin, TRPA1 has been found in several cell types including melanocytes, keratinocytes, and fibroblasts (Anand et al. 2008; Atoyan et al. 2009; Tsutsumi et al. 2010), and therefore, it has been hypothesized that TRPA1 plays a role in the regulation of keratinocyte differentiation and inflammation in the skin. TRPA1 has been found in epithelial cells lining the urinary tract (Streng et al. 2008; Gratzke et al. 2010) in addition to sensory fibers innervating the urinary tract (Andrade et al. 2006); therefore, TRPA1 may play a role in urinary micturition. It has also been found that TRPA1 is expressed in vascular endothelial (Gratzke et al. 2009; Qian et al. 2013) and in basal/subepithelial cells of the human prostate gland (Gratzke et al. 2010). Beta islet cells of the pancreas have been shown to express TRPA1, and activation of TRPA1 in these cells results in insulin release (Cao et al. 2012). Astrocytes in the brain appear to express TRPA1, and astrocyte TRPA1 channels appear to play an important role in calcium homeostasis and the regulation of the GABA transporter GAT-3 (Shigetomi et al. 2012), albeit at relatively lower lever than that of DRG neurons. As TRPA1 antagonists are developed for treating pain and other disorders, close examination of potential on-target side effects resulting from the expression of TRPA1 outside of the targeted tissue (e.g., DRG and TG) should be considered.

## Modulation of TRPA1

### Endogenous agonists

**Fig. 2:**
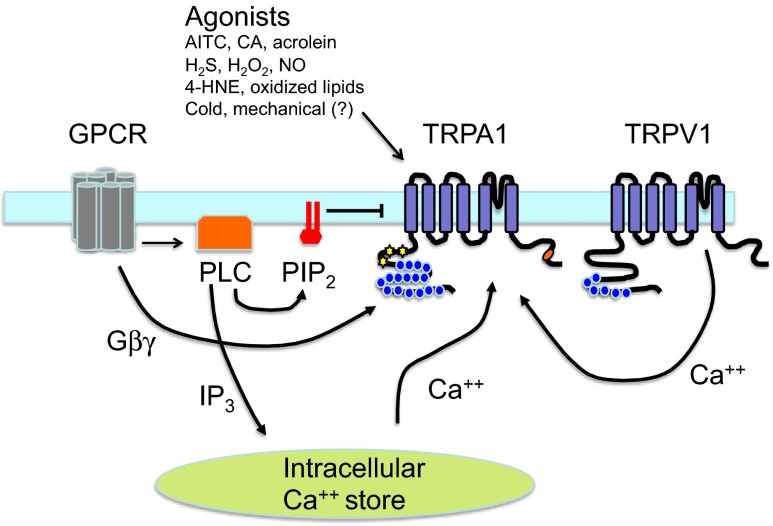
Modulation of TRPA1. TRPA1 is modulated by in multiple ways and represents an integrator of nociceptive signals. Agonists bind to the channel (most but not all within the N-terminal domain) and result in direct activation. PIP_2_ functions as an endogenous inhibitor which is removed by GPCR-dependent activation of PLC. PLC also liberates IP_3_, resulting in release of Ca^++^ from intracellular stores. Ca^++^, either from stores or from influx through other channels such as TRPV1, positively modulates TRPA1 via an intracellular binding site which has not been fully elucidated. Cold and mechanical forces can also activate TRPA1 via mechanisms that are not well understood

In order for TRPA1 to be regarded as a good target for pain and other disorders, it should be active in the context of some pathological state. The best-known activators of TRPA1, however, are a set of exogenous electrophilic agonists that form covalent adducts with cysteine and lysine residues contained within the N-terminal domain (Figs. 1 and 2). Examples of such covalent agonists are AITC from wasabi (Bandell et al. 2004; Jordt et al. 2004), cinnamaldehyde from cinnamon extracts (Bandell et al. 2004; Jordt et al. 2004), allicin from garlic extract (Bautista et al. 2005; Macpherson et al. 2005), acrolein in diesel exhaust (Bautista et al. 2006), and other noxious substances such as tear gases (Bessac et al. 2008). TRPA1 can therefore clearly function as a nociceptive chemosensor and may have evolved in part for this purpose. Endogenous agonists have also been found and have led to the idea that TRPA1 can be activated in the absence of exogenous agonists and potentially during certain pathological states. Examples of endogenous agonists are oxidized lipids such as 4-hydroxy-2-nonenal (4-HNE) (Taylor-Clark et al. 2008a; Trevisan et al. 2014a), 4-oxo-nonenal (Andersson et al. 2008), 5,6-eposyeicosatrienoic acid (5-6-EET) (Sisignano et al. 2012), 15-deoxy-∆12,14-prostaglandin J2 (Andersson et al. 2008; Cruz-Orengo et al. 2008), and 8-iso-prostaglandin A2 (Taylor-Clark et al. 2008b), nitrated lipids such as nitrooleic acid (9-OA-NO_2_) (Taylor-Clark et al. 2009), general long-chain polyunsaturated fatty acids (Motter and Ahern 2012), and small endogenous reactive molecules such as H_2_O_2_ (Andersson et al. 2008). In this manner, TRPA1 may be viewed as a sensor for oxidative stress since endogenous ligands can accumulate and activate the channel directly, thereby contributing to inflammatory pain in a TRPA1-dependent manner. Oxidative stress has also been proposed to play an important role in several rodent models of neuropathic pain (Kim et al. 2004; Naik et al. 2006), and recently, TRPA1 has been found in some cases to play an important role in linking the presence of oxidative stress to inflammatory and neuropathic pain (Trevisan et al. 2013a; Trevisan et al. 2013b; Trevisan et al. 2014b). Additionally, methylglyoxal, a reactive metabolite that accumulates intracellularly in diabetes (Brownlee 2001; Nakayama et al. 2008) and chronic kidney disease (Nakayama et al. 2008), is able to directly activate TRPA1 resulting in a painful neuropathy in mice (Eberhardt et al. 2012; Andersson et al. 2013). It is therefore possible that methyglyoxal contributes to diabetic neuropathic pain and the neuropathic pain present in patients with chronic kidney disease. Another classes of potentially physiologically relevant endogenous TRPA1 agonists are the small endogenous gasotransmitters NO (Takahashi et al. 2008) and H_2_S (Andersson et al. 2012). While these have been proposed to activate TRPA1 in vivo, the concentrations necessary to induce TRPA1 activation are higher than would likely occur physiologically. Recently, nitroxyl anion (HNO), which can be formed via chemical reaction between NO and H_2_S, has been shown to directly activate TRPA1 at physiologically relevant concentrations and may play an important role in local and systemic blood flow control (Eberhardt et al. 2014). This observation may open new therapeutic strategies for targeting cardiac failure but also indicates new potential adverse effects with TRPA1 blockade.

### Calcium

Intracellular calcium can modulate TRPA1 by both directly activating the channel as well as enhancing activation by agonists (e.g., AITC, cinnamaldehyde, and icilin) (Doerner et al. 2007; Zurborg et al. 2007; Wang et al. 2008b) (Fig. 2). In fact, activation by icilin may require the presence of elevated intracellular calcium (Doerner et al. 2007). Additionally, calcium influx is also able to rapidly inactivate the channel. Inactivation may occur at significantly higher local intracellular calcium concentrations than is required for calcium-dependent enhancement of activation (Wang et al. 2008b). At the molecular level, the mechanisms for such actions are not entirely understood. Calmodulin (CaM) appears not to be involved based on experiments co-expressing a dominant-negative CaM mutant and CaM antagonists (Doerner et al. 2007; Zurborg et al. 2007). An EF-hand-like region was identified within the ankyrin repeat of the N-terminal domain, but mutagenesis to disrupt the EF-hand resulted in inconsistent results. Specifically, mutation of the proposed essential EF-hand residue (D468A) did not alter calcium sensitivity (Doerner et al. 2007; Zurborg et al. 2007); the mutation of a nearby residue (L474A) was found to eliminate calcium sensitivity by one group (Doerner et al. 2007), but this result could not be reproduced by another group (Wang et al. 2008b). Adding more complexity to the matter, a third group identified an separate domain within N-terminal ankyrin repeat region (outside the EF-hand-like region) as essential for calcium-dependent inactivation (Cordero-Morales et al. 2011), while a fourth group suggested that acidic residues in the C-terminal domain form a calcium binding site similar to the calcium binding site present in BK potassium channels (Sura et al. 2012) (Fig. 2).

### Protons

Rodent DRG neurons are sensitive to low pH produced during tissue acidosis due to the presence of H^+^-sensitive ion channels such as TRPV1 and ASIC channels. Interestingly, it has recently been shown that human TRPA1, but not rodent TRPA1 or even rhesus monkey TRPA1, can be directly activated by protons (de la Roche et al. 2013). Proton activation occurs over a pH range from 6.0 to 7.0 with a midpoint of around pH 6.5, which is within the physiological pH range that would be expected to occur during tissue acidosis. It is currently unclear whether this observed pH dependence of human TRPA1 plays an important role in vivo.

### GPCR modulation

G protein-coupled receptors (GPCRs) such as the bradykinin receptor, the protease-activated receptor 2 (PAR2), the bile acid receptor TGR5, the thymic stromal lymphopoietin (TSLP) receptor, and the MAS-related GPCRs MrgprA3 and MrgprC11 have been shown to modulate the function of TRPA1 in cultured DRG neurons (Bandell et al. 2004; Dai et al. 2007; Wang et al. 2008a; Wilson et al. 2011; Wilson et al. 2013b; Lieu et al. 2014) (Fig. 2). Activation of these GPCRs results in the direct activation of TRPA1 and/or sensitization of TRPA1 to agonists such as AITC, cinnamaldehyde, and likely endogenous agonists. Activation of phospholipase C (PLC) appears to be required for such GPCR-mediated TRPA1 sensitization (with the exception of MrgprA3), though in the case of bradykinin receptor, protein kinase A (PKA) may play a role as well (Wang et al. 2008a). PLC likely sensitizes TRPA1 by removal of the inhibitory effect of PIP2 (Dai et al. 2007; Wang et al. 2008a) and by IP3-dependent release of calcium from intracellular stores (Jordt et al. 2004) (Fig. 2). Protein kinase C (PKC) appears not to play a role in bradykinin-dependent modulation of TRPA1 since neither GF109203X, a potent inhibitor of PKC, nor phorbol 12-myristate 13-acetate (PMA), a well-known PKC activator, alters bradykinin-mediated TRPA1 sensitization (Wang et al. 2008a). Nonetheless, PKC seems necessary for TGR5-dependent activation of TRPA1 (Lieu et al. 2014). MrgprA3 is activated by chloroquine and involved in histamine-independent itch. It signals through Gβγ, as gallein, a small-molecule inhibitor of Gβγ, markedly reduced chloroquine-evoked calcium influx in cultured DRG neurons, whereas U73122, a PLC inhibitor, had no effect (Wilson et al. 2011). A similar Gβγ dependence has been observed in the case of TGR5-dependent TRPA1 activation as well (Lieu et al. 2014).

### Mechanical sensitivity

Soon after its discovery, TRPA1 was proposed as a mechanosensor since its long ankyrin repeat could form a spring-like structure to sense mechanical forces (Howard and Bechstedt 2004; Sotomayor et al. 2005). In fact, it was brought forward early on as a candidate for the long sought-after tip-link mechanotransducer channel in hair cells of the inner ear (Corey et al. 2004). However, this idea was dismissed due to normal hair cell function and auditory function in TRPA1 KO mice (Bautista et al. 2006; Kwan et al. 2006). Direct mechanical stimulation of DRG neurons evokes currents that can be characterized as rapidly adapting, intermediate-adapting, and slowly adapting. Analysis of such currents from DRGs obtained from either WT and TRPA1 KO mice has yielded inconsistent results with one group showing a complete loss of slow-adapting currents, but not intermediate- or rapidly adapting currents from IB4-negative DRG neurons (Vilceanu and Stucky 2010), and another group showing a small but significant effect on intermediate-adapting currents from small-diameter DRGs with no effect on slowly adapting currents. Further evidence for direct mechanosensitivity of TRPA1 has been obtained from TRPA1-expressing HEK293 cells where membrane shrinkage (via hypertonicty) and membrane curvature (via trinitrophenol) appear to activate TRPA1 (Hill and Schaefer 2007; Zhang et al. 2008). Most recently, it was shown that lipopolysaccharide (LPS), an abundant outer membrane glycolipid released by Gram-negative bacteria, activates endogenously expressed and heterologously expressed TRPA1 channels (Meseguer et al. 2014). The activation depends on the shape of membrane-anchoring lipid moiety of LPS, indicating that perturbation of the plasma membrane may underline channel activation. Hypertonicity, trinitrophenol, and LPS may serve to sensitize TRPA1 channel despite that fact that their direct effects on TRPA1 are relatively small compared to those of electrophilic agonists (e.g., AITC). More research in this area will be needed to better understand whether TRPA1 is indeed acting as a direct mechanosensor.

In vitro skin–nerve preparation recordings indicate that TRPA1 gene KO and treatment with the TRPA1 antagonist HC-030031 can reduce mechanically induced action potential firing in dermal C-fibers (Kerstein et al. 2009; Kwan et al. 2009). A similar reduction in action potential frequency was observed in recordings of mechanosensitive visceral afferents from the colon (Brierley et al. 2009). Furthermore, the increased mechanical sensitivity observed in the skin–nerve preparation following CFA could be reversed by HC-030031 (Lennertz et al. 2012). In vivo electrophysiology recordings showed that A-967079 reduced the responsiveness of spinal wide dynamic range (WDR) and nociceptive-specific neurons to high-intensity mechanical stimuli in naïve and inflamed rats (McGaraughty et al. 2010). However, A-967079 only affected low-intensity mechanical stimuli-evoked responses in inflamed but not naïve rats.

Behavioral studies of acute mechanical nociception of TRPA1 KO mice have shown rather inconsistent results. Two early studies revealed no apparent deficit in acute mechanical nociception in KO mice (Bautista et al. 2006; Petrus et al. 2007), while deficits were detected in other studies (Kwan et al. 2006; Garrison and Stucky 2014; Minett et al. 2014). In particular, Minett et al. compared KO mouse strains of Nav1.3, Nav1.7, Nav1.8, Nav1.9, and TRPA1 and found that only TRPA1 KO mice had a defect in mechanical nociception as measured using the Randall-Selitto test applied to the paw (Minett et al. 2014). Interestingly, TRPA1 KO mice showed normal mechanical nociception using the same test applied to the tail (where both Nav1.7 and Nav1.8 KO mice displayed a clear defect). Finally, the deficit in acute mechanical sensitivity observed in TRPA1 KO mouse should be carefully interpreted since a recent study showed a significant reduction in intraepidemal nerve fiber density in TRPA1 KO compared to wild-type mice (Andersson et al. 2013).

### Cold sensitivity

Activation of TRPA1 by cold (temperature <17 °C) has been shown by some groups (Bandell et al. 2004; Sawada et al. 2007; Karashima et al. 2009; del Camino et al. 2010; Lehto et al. 2013) but disputed by others (Jordt et al. 2004; Zurborg et al. 2007; Cordero-Morales et al. 2011). Efforts to resolve the discrepancy were confounded by differences in experimental procedures, including methodologies (Ca^2+^ imaging, whole-cell or single-channel recordings), ionic conditions (with or without extracellular Ca^2+^), and clones from different species (human, rat, and mouse). In a recent study, TRPA1 from four different mammalian species were characterized under the same experimental conditions (Chen et al. 2013). In Ca^2+^-influx assays as well as whole-cell and single-channel recordings, cold activates rat and mouse TRPA1, albeit with lower efficacy compared to AITC (~40 % open-probability relative to AITC). In contrast, neither human nor rhesus monkey TRPA1 could be activated by cold. These findings are in line of most of the literature data and suggest that previous discrepancies could be due to species differences. However, cold activation of human TRPA1 has been observed in whole-cell recordings by some groups (Bandell et al. 2004; Kremeyer et al. 2010), and human TRPA1 reconstituted into lipid bilayers is intrinsically cold-sensitive (Moparthi et al. 2014). Therefore, whether TRPA1 is a cold-sensitive channel is not formally settled. At behavioral level, at least five studies using TRPA1 KO mice produced conflicting results. Two studies found that TRPA1 KO mice retained normal responses in cold plate and acetone-evoked evaporative cooling tests and upon cold stimulation maintained normal c-fos expression (a marker for neural activity) (Bautista et al. 2006; Knowlton et al. 2010). A third study reported that female, but not male, KO mice had reduced cold sensitivity (Kwan et al. 2006). A fourth study reported that TRPA1 KO mice had no deficit in acute cold sensation but lost the jumping behavior when challenged with prolonged cold exposure (Karashima et al. 2009). The fifth study found no difference in cold sensitivity between wild-type and KO mice but found that cold enhanced the effects of AITC, thereby contributing to AITC-induced cold allodynia (del Camino et al. 2010). In pharmacology studies using TRPA1 antagonists, A-967079 did not affect acute cold sensing in uninjured mice but attenuated cold allodynia in neuropathic rats (Chen et al. 2011a). Similarly, HC-030031 was shown to attenuate cold hyperalgesia in CFA (inflammatory), spared never injury (SNI, neuropathic), and paclitaxel-mediated cold hyperalgesia (del Camino et al. 2010; Materazzi et al. 2012). Additionally, TRPA1-specific antisense oligodeoxynucleotides were efficacious in attenuating cold allodynia in inflammatory and neuropathic pain models (Obata et al. 2005). Taken together, these results suggest that TRPA1 might not play a significant role in acute cold sensation but may be involved in cold allodynia under disease states where endogenous TRPA1 activation mechanisms are present.

Intriguingly, TRPA1 knockout mice showed higher tolerance to heat, higher heat threshold of cutaneous C-mechano-heat-sensitive fibers, and less heat-induced CGRP release (Hoffmann et al. 2013). Also HC-030031 was found to decrease heat hyperalgesia in the paclitaxel model of chemotherapy-induced neuropathic pain (Chen et al. 2011b). These effects might be due to TRPA1 sensitization, instead of direct activation by heat. Unlike their counterparts in invertebrate and ancestral vertebrates, mammalian TRPA1 channels are not activated by heat directly (Chen et al. 2013).

### Physiological and pathological role of TRPA1

#### Pain

Several lines of evidence suggest that TRPA1 is involved in pain sensation. TRPA1 is expressed in sensory neurons and co-localized with pain markers such as TRPV1 and bradykinin receptors (Obata et al. 2005; Bautista et al. 2006). Its expression is increased in animal models of inflammatory and neuropathic pain and in human avulsion-injured DRG neurons (Obata et al. 2005; Anand et al. 2008). TRPA1 agonists evoke neurotransmitter release, pain, and inflammation in rodents and humans (Namer et al. 2005; Bautista et al. 2006; Kwan et al. 2006), and endogenous agonists such as 4-HNE are elevated under human pathologies (Morquette et al. 2006). Gene KO attenuates agonist sensitivity, and antagonist treatment attenuates pain in several animal models (Bautista et al. 2006; Kwan et al. 2006; McNamara et al. 2007; Petrus et al. 2007; Eid et al. 2008; Chen et al. 2011a). Recently, a TRPA1 gain of function mutation was linked to human congenital pain condition called familial episodic pain syndrome (FEPS) (Kremeyer et al. 2010). Based on data in the literature, TRPA1 has been suggested to play a role in many sensory modalities, including chemical nociception, mechanical nociception, and cold nociception.

Among various sensory modalities, the best-established and least controversial is chemical nociception. TRPA1 detects a variety of exogenous noxious chemicals such as AITC, cinnamaldehyde, allicin, and acrolein. The response of DRG neurons to these compounds and the nocifensive behaviors they elicit have been rigorously demonstrated to be TRPA1-dependent after TRPA1 KO mice became available in 2006 (Bautista et al. 2006; Kwan et al. 2006). The same level of rigor has been applied to identify endogenous agonists as well, clearly establishing that endogenous agonist-dependent DRG calcium influx and nocifensive behaviors are TRPA1 dependent. Less clear, however, is the role that these endogenous activators play in inflammatory and/or neuropathic pain states. For example, while injection of the glucose metabolite methlyglyoxal (MG) results in a TRPA1-dependent sensory neuropathy in mice (Eberhardt et al. 2012; Andersson et al. 2013) and while MG is elevated in patients with diabetic neuropathy, it has not yet been demonstrated that MG or TRPA1 plays a causative role of any kind in diabetic neuropathy. Similarly, while many other endogenous activators of TRPA1 (e.g., 4-HNE and acrolein) have been found to be sufficient in causing pain behavior in rodents, there is currently no evidence that these molecules play a causative role in any form of inflammatory or neuropathic pain.

**Fig. 3:**
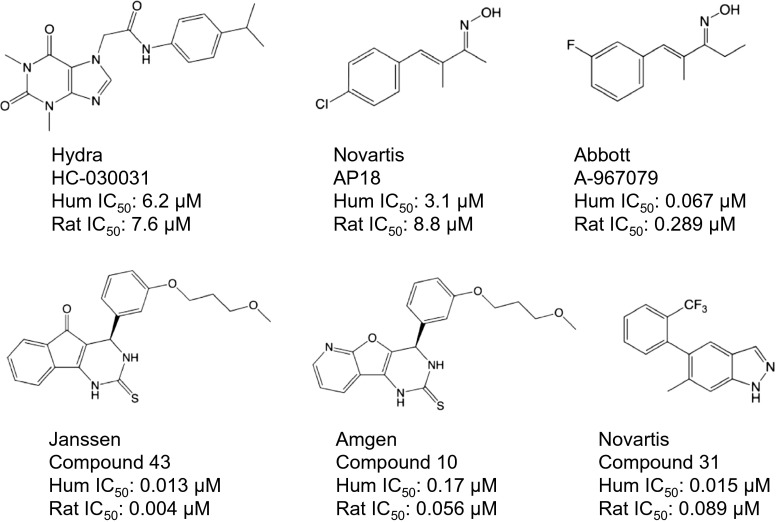
Examples of TRPA1 antagonists. HC-030031, AP18, and A-967079 are currently the most commonly used TRPA1 tool antagonists for studying TRPA1 biology in vivo and in vitro. A newer generation of TRPA1 antagonists (*lower row*) have recently been developed that offer better potency on the rodent TRPA1 channels as well as significantly improved PK properties compared to earlier generation compounds

TRPA1 has also been proposed to play a role in mechanical allodynia following induction of inflammatory and neuropathic pain, indicating that TRPA1 might be a potential target for novel chronic pain drugs. Early reports on the phenotype of the TRPA1 KO mouse indicated that there is little if any defect in mechanical allodynia following either CFA-induced inflammatory pain or nerve injury-induced mechanical allodynia (Kwan et al. 2006; Petrus et al. 2007). However, more recent TRPA1 KO experiments appear to show a major defect in CFA-induced inflammatory pain in the TRPA1 KO mouse, however, especially in older mice (Garrison and Stucky 2014). Also, while Petrus et al. 2007 failed to find a defect in the CFA model in the TRPA1 KO mouse, they were able to show that the TRPA1 blocker AP-18 was able to reverse CFA-induced inflammatory pain in WT mice but not in TRPA1 KO mice, indicating that indeed TRPA1 contributes to CFA-induced mechanical allodynia and perhaps some form of compensation occurs in the TRPA1 KO mouse allowing a TRPA1-independent CFA-response in these animals. Other TRPA1 antagonists such as HC-030031 have shown similar attenuation of mechanical allodynia in the CFA model (Petrus et al. 2007; Eid 2009). Intrathecally administered Chembridge-5861528 attenuated mechanical hypersensitivity in spinal nerve ligation model and capsaicin-induced secondary hyperalgesia model (Wei et al. 2011). However, some newer generation TRPA1 antagonists with more robust exposure (free plasma concentration vs. in vitro IC_50_) (Fig. 3) were not effective in these models despite showing robust inhibition of AITC-induced nocifensive behavior. For example, A-967079 at IC_85_ coverage did not attenuate mechanical hyperalgesia in CFA, chronic constriction injury, or spinal nerve ligation models (Chen et al. 2011a). AMG0902 at an even higher drug exposure (fourfold over IC_90_ coverage) had only a modest effect on mechanical allodynia in CFA model and no effect in the spinal nerve ligation (SNL) model (Lehto et al. 2013). On the other hand, potent inhibitors from Glenmark Pharmaceutical Ltd. as well as the recently reported potent inhibitor from Novartis (Novartis compound 31 in Fig. 3) did appear to show efficacy in the CFA model (Rooney et al. 2014).

Several groups have demonstrated a role for TRPA1 in rodent models of chemotherapy-induced painful neuropathy (CIPN) and painful diabetic neuropathic (PDN). These two forms of neuropathic pain are of particular interest since they can be directly studied in early proof-of-concept clinical trials in humans. As shown using either TRPA1 KO mice or the TRPA1 antagonist HC-030031, TRPA1 appears to contribute significantly to paclitaxel-induced mechanical allodynia (Materazzi et al. 2012) as well as bortezomib- and oxaliplatin-induced mechanical allodynia (Trevisan et al. 2013a) in mice. Strikingly, block of TRPA1 during the 5-h period encompassing the time of bortezomib dosing appears to completely prevent the development of neuropathic pain in this model (Trevisan et al. 2013a). In the case of DPN, no published data exists showing whether or not the development of diabetic neuropathic pain is impaired in TRPA1 KO mice. However, intrathecal injections of the TRPA1 antagonist CHEM-5861528 have been found to reverse mechanical allodynia in streptozotocin-induced diabetic rats (Wei et al. 2009; Wei et al. 2010) and may prevent diabetes-induced loss of nociceptor nerve endings in the skin (Koivisto et al. 2012). More recently, Glenmark Pharmaceuticals Ltd. reported that GRC 17536, a peripheral acting TRPA1 antagonist, exhibited efficacy in a phase 2a proof-of-concept study in patients with painful diabetic neuropathy.

TRPA1 is present in nociceptive fibers innervating internal organs (e.g., colon and pancreas) and implicated in visceral pain and inflammation. When activated during inflammation, these fibers carry visceral pain signals and also release neuropeptides such as substance P and CGRP, resulting in neurogenic inflammation. Desensitization of TRPV1 receptor-expressing C-fibers using treatment with either resiniferatoxin (RTX) or capsaicin results in significantly reduced severity of dextran-sulfate-sodium (DSS)-induced colitis in mice (Kihara et al. 2003; Engel et al. 2012), indicating that TRPV1-positive nociceptor fibers are involved in this model of colitis. Furthermore, DSS-induced colitis was found to be ameliorated in both CGRP KO mice and substance P KO mice (Engel et al. 2012). TRPV1 itself does not appear to play a role in either DSS-induced colitis or trinitrobenzene-sulfonic-acid (TNBS)-induced colitis as these models are not altered in the TRPV1 KO mouse (Engel et al. 2011; Engel et al. 2012). However, both DSS-induced and TNBS-induced colitis were significantly reduced in TRPA1 KO mice or WT mice treated with the TRPA1 antagonist HC-030031. Similar data have been obtained in the case of caerulein-induced pancreatitis, where TRPA1 antagonists and genetic removal of TRPA1 (TRPA1 KO mouse) have been shown to reduce the extent of inflammation and pain in this model (Ceppa et al. 2010; Schwartz et al. 2011; Schwartz et al. 2013).

Spinal activation of TRPA1 may contribute to pain and represents a target for pharmacological intervention (Wei et al. 2011). However, a recent study suggests that spinal TRPA1 activation may underlie the antinociceptive effect of acetaminophen (Anderson et al. 2011). It was shown that reactive metabolites of acetaminophen can directly activate TRPA1 at the central terminals of primary sensory neurons, causing inactivation of voltage-gated Ca^2+^ and Na^+^ channels and thereby reducing neurotransmitter release and neuronal excitability. The antinociceptive effect of spinal TRPA1 activation does not appear limited to covalent TRPA1 agonists, as Δ^9^-tetrahydrocannabiorcol, a noncovalent agonist, is also antinociceptive in the hot plate test. Thus, spinal activation of TRPA1 can be either nociceptive or antinociceptive, perhaps depending on the degree of TRPA1 activation. Therefore, it is possible that both antagonists and agonists of TRPA1 may have utility for pain relief.

#### Respiratory diseases

TRPA1 is expressed in primary sensory neurons innervating the airways where it acts as a chemosensor for airway irritants such as acrolein, ozone, isocyanate, tear gas, and chlorine (Andre et al. 2008; Bessac et al. 2008; Bessac et al. 2009). TRPA1 activation leads to pain and reflex (sneezing, cough, and respiratory depression and avoidance), therefore serves as a protection to limit or eliminate irritant exposure. TRPA1 can also be activated chronically in chronic cough, asthma, chronic obstructive pulmonary disease (COPD), and rhinitis, wherein endogenous TRPA1 ligands (e.g., 4HNE, H_2_O_2_) and pro-inflammatory mediators (e.g., bradykinin and nerve growth factors) are elevated (Dolovich et al. 1970; Winterbourn and Kettle 2000). These ligands activate TRPA1 directly (e.g., 4HNE and H_2_O_2_), indirectly (bradykinin), or by increasing TRPA1 surface expression (nerve growth factor). Reciprocally, heightened TRPA1 function leads to neurotransmitter release and promotes neurogenic inflammation (Andre et al. 2008; Caceres et al. 2009).

The role of TRPA1 in airway pathologies has been corroborated by studies using the TRPA1 KO mouse and TRPA1 antagonists. In wild-type mice, airway exposure to hypochlorite or H_2_O_2_ evoked respiratory depression as manifested by a reduction in breathing frequency and increase in end expiratory pause, both of which were attenuated in TRPA1 KO mice (Bessac et al. 2008). In an ovalbumin-induced mouse asthma model, gene KO and treatment with HC-030031 reduced the induction of cytokines, chemokines, neurotransmitters, as well as leukocyte infiltration and airway hyperactivity (Caceres et al. 2009). In the guinea pig, acrolein, crotonaldehyde, and extracts from cigarette smoke induced neurotransmitter release, tracheal plasma extravasation, and bronchi contraction. These effects could be attenuated by HC-030031 (Andre et al. 2008). These results are certainly encouraging, although it is worth noting that chronic respiratory diseases are multifaceted disorders, often involving airway damage and tissue remodeling. It remains to be demonstrated whether TRPA1 antagonists are capable of reversing disease progression and outperforming current standard of care such as anti-inflammatory drugs and bronchodilators.

#### Itch

Recent studies suggest that TRPA1 is involved in mediating histamine-independent itch (Wilson et al. 2011; Wilson et al. 2013a). The antimalarial drug chloroquine and the endogenous pruritogen BAM8-22 induce itch through Mas-related G protein-coupled receptors (MrgprA3 and MrgprC11, respectively), and their effect is mediated through TRPA1. In lesioned skin of human atopic dermatitis patients, TRPA1 expression is increased in dermal afferent fibers, dermal cells, and mast cells (Oh et al. 2013). In a mouse model of atopic dermatitis generated by overexpression of IL-13, TRPA1 expression is also increased in afferent fibers, dorsal root ganglia, and mast cells in a manner similar to that observed in human patients (Oh et al. 2013). Furthermore, HC-030031 and genetic deletion of mast cells attenuated itch-scratching behaviors. In oxazolone-induced contact dermatitis models, TRPA1 KO and HC-030031 decreased pro-inflammatory cytokines, T cell infiltration, dermatitis score, and edema, indicating that TRPA1 may play a central role in inflammation and pruritus (Liu et al. 2013).

#### Antagonist tools

An increasing number of TRPA1 antagonists have been disclosed, and only a few examples are listed here (Fig. 3). HC-030031, a xanthine alkaloid discovered by Hydra Bioscience, has been the most widely used in the literature as a tool for studying TRPA1-mediated biology (McNamara et al. 2007; Eid et al. 2008). HC-030031 inhibits human and rat TRPA1 with IC_50_ of 6.2 and 7.6 μM, respectively (McNamara et al. 2007; Bianchi et al. 2012). It is selective against several TRP channels (IC_50_ >10 or 20 μM). However, when tested in radioligand binding assays at 10 μM concentration, HC-030031 showed activity against several proteins including sodium channels (40 %) and sigma receptors (37 %) (Chen et al. 2011a). In addition, HC-030031 has high clearance (27 ml/kg/min), low exposure (Cmax = 355 ng/ml following a 12-mg/kg dose), a short half-life (32 min), and high plasma protein binding (90 %) in rats (Rech et al. 2010). With such poor pharmacokinetic (PK) properties, it is unlikely that HC-030031 can reach unbound concentrations above the TRPA1 IC_50_ even at very high doses. Nonetheless, it exhibited efficacy in CFA, SNL, and other pain models (McNamara et al. 2007; Petrus et al. 2007; Eid et al. 2008). The efficacy of HC-030031 has not been conclusively attributed to the block of TRPA1 by showing, for example, that efficacy is lost in the TRPA1 KO mouse, and therefore, results obtained with this low-potency compound should be interpreted with caution.

Recently, Abbott, Amgen, AstraZenica, Glenmark, Hydra Bioscience, Janssen, Merck, and other companies have disclosed additional TRPA1 antagonists with improved potency, selectivity, and pharmaceutical properties (Fig. 3). Pharmacokinetic and efficacy data have been described for A-967079 (Abbott), Compound 10 (Amgen), and Compound 31 (Novartis) (Chen et al. 2011a; Copeland et al. 2014; Rooney et al. 2014). Compound 10, an azabenzofuran analog, inhibits human and rat TRPA1 with IC_50_ of 0.17 and 0.045 μM, respectively, and has favorable PK properties allowing robust unbound plasma exposure (14-fold over rat IC_50_ when dosed at 100 mg/kg). This compound was highly efficacious in the AITC-flinch model, but its efficacy in other pain models was not reported (Copeland et al. 2014). This new generation of TRPA1 antagonists will be valuable in exploring TRPA1 function and therapeutic utility and will hopefully replace the use of older TRPA1 tool compounds.

Beside small molecules, monoclonal antibodies (mAbs) have been generated using a cell immunogen approach (Lee et al. 2014). The most potent mAb, 2B10, inhibited activation by AITC (IC_50_, 260 nM), cold (IC_50_, 90 nM), and hypertonicity (IC_50_, 350 nM). 2B10 also had similar effect on mouse TRPA1, and therefore, it likely targets a domain conserved in human and mouse channels, probably in the extracellular pore loop. Similar to pore-blocking antibodies for other ion channels, the inhibition of TRPA1 plateaued at 70 %. 2B10 might be a potentially useful tool if it can get access to relevant tissues.

#### Species differences

The electrophile sensitivity of TRPA1 is conserved across species (e.g., fruit fly, lizard, snake, chicken, mouse, and humans), indicating electrophile detection is a core function of TRPA1. On the other hand, TRPA1 also exhibits evolutionary divergence in temperature sensitivities, from being heat-sensitive in invertebrates/ancestral vertebrates (fruit fly, rattle snake, lizard, and frog) (Viswanath et al. 2003; Hamada et al. 2008; Kwon et al. 2008; Gracheva et al. 2010; Kang et al. 2012), to being cold-sensitive in rodents (Story et al. 2003; Chen et al. 2013), and to being temperature-insensitive in primates (Jordt et al. 2004; Chen et al. 2013). The evolutionary divergence in thermo-sensitivity indicates the likelihood that TRPA1 from different species (e.g., human and rats) may have different functions. At the amino acid level, human and rat TRPA1 are poorly conserved (79 % identical, compared to 94 % for TRPM8 and 86 % for TRPV1 for examples), which may lead to qualitative or quantitative differences in sensitivity to ligands. Additionally, other cross-species variations (metabolic mechanisms, transduction pathways, and animal physiology) can lead to species difference of TRPA1 function.

Species difference in response to pharmacological agents has been well demonstrated for TRPA1 (Bianchi et al. 2012). For example, thioaminols and caffeine activate rodent channels but block the human channel (Klionsky et al. 2007; Chen et al. 2008; Nagatomo et al. 2010). Menthol activates the human channel at high concentration but blocks rodent channels (Xiao et al. 2008). In high throughput screening and medicinal chemistry efforts, we and others have found that a majority of human TRPA1 antagonists have reduced potency, or act as agonists, on the rodent TRPA1 channels. Such compounds could not be advanced in the drug discovery process due to the need to use rodents as preclinical species. Recently, we showed that monkey and human TRPA1 share similar pharmacology and therefore monkey could potentially serve as a surrogate species (Bianchi et al. 2012). However, the practical use of monkeys is hindered by high cost, low throughput, and the limited number of monkey disease models available. The generation of humanized transgenic animals (i.e., knocking-in of human TRPA1) could be a valuable alternative path forward. In all, species difference is a serious problem and requires creative solutions.

#### Potential adverse effects

Although TRPA1 is predominantly expressed in sensory neurons, it is also expressed in other tissues. Therefore, targeting TRPA1 could have unintended adverse effects. TRPA1 KO mince appear normal in appearance, reproduction, and auditory function (Bautista et al. 2006; Kwan et al. 2006), although physical hyperactivity was recently reported (Bodkin et al. 2014). Unlike TRPV1 or TRPM8, TRPA1 is not involved in body temperature regulation at basal level or under cold challenge (Chen et al. 2011a; de Oliveira et al. 2014), and therefore, TRPA1 antagonists should be devoid of the adverse effects associated with TRPV1 antagonists (hyperthermia) or TRPM8 antagonists (hyperthermia).

One potential safety concern for TRPA1 antagonists is the deficit in sensing environmental irritants. Activation of TRPA1 by acrolein, ozone, and chlorine leads to pain, mucus secretion, sneezing, cough, and respiratory depression (Bautista et al. 2006; Andre et al. 2008; Bessac et al. 2008; Kwan et al. 2009). These nociceptive and behavioral responses help limit or eliminate irritant exposure, therefore protecting the body from damage. As an example, chlorine is widely used as a domestic (disinfection) and industry product with an annual consumption of 15 million tons in the USA. While most individuals can react quickly to avoid severe exposure, injuries do occur in the form of pulmonary edema, restrictive lung diseases, and obstructive disease. Airway exposure of chlorine results in reduced breathing frequency and increased end expiratory pause in wild-type mice, but such protective reflexes are absent in KO mice (Bessac et al. 2008). Will TRPA1 antagonists cause deficits in sensing and avoiding irritants in humans? This question should be addressed during clinical development.

## Concluding remarks

In the TRP family and ion channel superfamily as a whole, TRPA1 distinguishes itself by its covalent activation mechanism and its role in sensing a wide range of noxious chemicals. TRPA1 is also one of the few ion channels implicated in pain by human genetics. A large body of evidence supports the therapeutic utility of TRPA1 antagonists for pain, respiratory, itch, and other diseases. Additionally, TRPA1 antagonists appear not to cause significant safety concerns thus far. As such, TRPA1 has replaced TRPV1 as one of the most sought-after therapeutic targets for novel pain drugs. In September 2014, Glenmark announced that GRC 17536, a peripherally acting selective TRPA1 antagonist, exhibited efficacy in a phase 2a proof-of-concept study in patients with painful diabetic neuropathy. Despite this clear progress, there are many unanswered questions. Is TRPA1 a receptor for noxious cold? Does it play a role in mechosensation? What are the endogenous ligands? Do TRPA1 KO mice tell the full story? Do TRPA1 have cross-species difference in physiological and pathological function? What are the best therapeutic indications for TRPA1 antagonists? Do TRPA1 antagonists cause deficits in sensing noxious irritants? Recognizing and addressing these questions will not only reveal the physiological function of TRPA1 but also pave the way for developing new therapies.

## References

[CR1] Anand U, Otto WR, Facer P, Zebda N, Selmer I, Gunthorpe MJ, Chessell IP, Sinisi M, Birch R, Anand P (2008). TRPA1 receptor localisation in the human peripheral nervous system and functional studies in cultured human and rat sensory neurons. Neurosci Lett.

[CR2] Anderson DA, Gentry, C, Lewis, S, Hogestatt, E, Zygmunt, P, Bevan S (2011) TRPA1 mediates acetaminophen (paracetamol) induced spinal antinociception. Soc Neurosci 1089 10.1038/ncomms1559 22109525

[CR3] Andersson DA, Gentry C, Moss S, Bevan S (2008). Transient receptor potential A1 is a sensory receptor for multiple products of oxidative stress. J Neurosci.

[CR4] Andersson DA, Gentry C, Bevan S (2012). TRPA1 has a key role in the somatic pro-nociceptive actions of hydrogen sulfide. PLoS One.

[CR5] Andersson DA, Gentry C, Light E, Vastani N, Vallortigara J, Bierhaus A, Fleming T, Bevan S (2013). Methylglyoxal evokes pain by stimulating TRPA1. PLoS One.

[CR6] Andrade EL, Ferreira J, Andre E, Calixto JB (2006). Contractile mechanisms coupled to TRPA1 receptor activation in rat urinary bladder. Biochem Pharmacol.

[CR7] Andre E, Campi B, Materazzi S, Trevisani M, Amadesi S, Massi D, Creminon C, Vaksman N, Nassini R, Civelli M, Baraldi PG, Poole DP, Bunnett NW, Geppetti P, Patacchini R (2008). Cigarette smoke-induced neurogenic inflammation is mediated by alpha, beta-unsaturated aldehydes and the TRPA1 receptor in rodents. J Clin Invest.

[CR8] Atoyan R, Shander D, Botchkareva NV (2009). Non-neuronal expression of transient receptor potential type A1 (TRPA1) in human skin. J Invest Derm.

[CR9] Bandell M, Story GM, Hwang SW, Viswanath V, Eid SR, Petrus MJ, Earley TJ, Patapoutian A (2004). Noxious cold ion channel TRPA1 is activated by pungent compounds and bradykinin. Neuron.

[CR10] Bautista DM, Movahed P, Hinman A, Axelsson HE, Sterner O, Hogestatt ED, Julius D, Jordt SE, Zygmunt PM (2005). Pungent products from garlic activate the sensory ion channel TRPA1. Proc Natl Acad Sci U S A.

[CR11] Bautista DM, Jordt SE, Nikai T, Tsuruda PR, Read AJ, Poblete J, Yamoah EN, Basbaum AI, Julius D (2006). TRPA1 mediates the inflammatory actions of environmental irritants and proalgesic agents. Cell.

[CR12] Bessac BF, Sivula M, von Hehn CA, Escalera J, Cohn L, Jordt SE (2008). TRPA1 is a major oxidant sensor in murine airway sensory neurons. J Clin Invest.

[CR13] Bessac BF, Sivula M, von Hehn CA, Caceres AI, Escalera J, Jordt SE (2009). Transient receptor potential ankyrin 1 antagonists block the noxious effects of toxic industrial isocyanates and tear gases. FASEB J.

[CR14] Bianchi BR, Zhang XF, Reilly RM, Kym PR, Yao BB, Chen J (2012). Species comparison and pharmacological characterization of human, monkey, rat, and mouse TRPA1 channels. J Pharmacol Exp Ther.

[CR15] Bodkin JV, Thakore P, Aubdool AA, Liang L, Fernandes ES, Nandi M, Spina D, Clark JE, Aaronson PI, Shattock MJ, Brain SD (2014). Investigating the potential role of TRPA1 in locomotion and cardiovascular control during hypertension. Pharmacol Res Perspect.

[CR16] Brierley SM, Hughes PA, Page AJ, Kwan KY, Martin CM, O’Donnell TA, Cooper NJ, Harrington AM, Adam B, Liebregts T, Holtmann G, Corey DP, Rychkov GY, Blackshaw LA (2009). The ion channel TRPA1 is required for normal mechanosensation and is modulated by algesic stimuli. Gastroenterology.

[CR17] Brownlee M (2001). Biochemistry and molecular cell biology of diabetic complications. Nature.

[CR18] Caceres AI, Brackmann M, Elia MD, Bessac BF, del Camino D, D’Amours M, Witek JS, Fanger CM, Chong JA, Hayward NJ, Homer RJ, Cohn L, Huang X, Moran MM, Jordt SE (2009). A sensory neuronal ion channel essential for airway inflammation and hyperreactivity in asthma. Proc Natl Acad Sci U S A.

[CR19] Cao DS, Zhong L, Hsieh TH, Abooj M, Bishnoi M, Hughes L, Premkumar LS (2012). Expression of transient receptor potential ankyrin 1 (TRPA1) and its role in insulin release from rat pancreatic beta cells. PLoS One.

[CR20] Ceppa E, Cattaruzza F, Lyo V, Amadesi S, Pelayo JC, Poole DP, Vaksman N, Liedtke W, Cohen DM, Grady EF, Bunnett NW, Kirkwood KS (2010). Transient receptor potential ion channels V4 and A1 contribute to pancreatitis pain in mice. Am J Physiol Gastrointest Liver Physiol.

[CR21] Chen J, Zhang XF, Kort ME, Huth JR, Sun C, Miesbauer LJ, Cassar SC, Neelands T, Scott VE, Moreland RB, Reilly RM, Hajduk PJ, Kym PR, Hutchins CW, Faltynek CR (2008). Molecular determinants of species-specific activation or blockade of TRPA1 channels. J Neurosci.

[CR22] Chen J, Joshi SK, DiDomenico S, Perner RJ, Mikusa JP, Gauvin DM, Segreti JA, Han P, Zhang XF, Niforatos W, Bianchi BR, Baker SJ, Zhong C, Simler GH, McDonald HA, Schmidt RG, McGaraughty SP, Chu KL, Faltynek CR, Kort ME, Reilly RM, Kym PR (2011). Selective blockade of TRPA1 channel attenuates pathological pain without altering noxious cold sensation or body temperature regulation. Pain.

[CR23] Chen Y, Yang C, Wang ZJ (2011). Proteinase-activated receptor 2 sensitizes transient receptor potential vanilloid 1, transient receptor potential vanilloid 4, and transient receptor potential ankyrin 1 in paclitaxel-induced neuropathic pain. Neuroscience.

[CR24] Chen J, Kang D, Xu J, Lake M, Hogan JO, Sun C, Walter K, Yao B, Kim D (2013). Species differences and molecular determinant of TRPA1 cold sensitivity. Nat Commun.

[CR25] Copeland KW, Boezio AA, Cheung E, Lee J, Olivieri P, Schenkel LB, Wan Q, Wang W, Wells MC, Youngblood B, Gavva NR, Lehto SG, Geuns-Meyer S (2014). Development of novel azabenzofuran TRPA1 antagonists as in vivo tools. Bioorg Med Chem Lett.

[CR26] Cordero-Morales JF, Gracheva EO, Julius D (2011). Cytoplasmic ankyrin repeats of transient receptor potential A1 (TRPA1) dictate sensitivity to thermal and chemical stimuli. Proc Natl Acad Sci U S A.

[CR27] Corey DP, Garcia-Anoveros J, Holt JR, Kwan KY, Lin SY, Vollrath MA, Amalfitano A, Cheung EL, Derfler BH, Duggan A, Geleoc GS, Gray PA, Hoffman MP, Rehm HL, Tamasauskas D, Zhang DS (2004). TRPA1 is a candidate for the mechanosensitive transduction channel of vertebrate hair cells. Nature.

[CR28] Cruz-Orengo L, Dhaka A, Heuermann RJ, Young TJ, Montana MC, Cavanaugh EJ, Kim D, Story GM (2008). Cutaneous nociception evoked by 15-delta PGJ2 via activation of ion channel TRPA1. Mol Pain.

[CR29] Dai Y, Wang S, Tominaga M, Yamamoto S, Fukuoka T, Higashi T, Kobayashi K, Obata K, Yamanaka H, Noguchi K (2007). Sensitization of TRPA1 by PAR2 contributes to the sensation of inflammatory pain. J Clin Invest.

[CR30] de la Roche J, Eberhardt MJ, Klinger AB, Stanslowsky N, Wegner F, Koppert W, Reeh PW, Lampert A, Fischer MJ, Leffler A (2013). The molecular basis for species-specific activation of human TRPA1 protein by protons involves poorly conserved residues within transmembrane domains 5 and 6. J Biol Chem.

[CR31] de Oliveira C, Garami A, Lehto SG, Pakai E, Tekus V, Pohoczky K, Youngblood BD, Wang W, Kort ME, Kym PR, Pinter E, Gavva NR, Romanovsky AA (2014). Transient receptor potential channel ankyrin-1 is not a cold sensor for autonomic thermoregulation in rodents. J Neurosci: Off J Soc Neurosci.

[CR32] del Camino D, Murphy S, Heiry M, Barrett LB, Earley TJ, Cook CA, Petrus MJ, Zhao M, D’Amours M, Deering N, Brenner GJ, Costigan M, Hayward NJ, Chong JA, Fanger CM, Woolf CJ, Patapoutian A, Moran MM (2010). TRPA1 contributes to cold hypersensitivity. J Neurosci.

[CR33] Doerner JF, Gisselmann G, Hatt H, Wetzel CH (2007). Transient receptor potential channel A1 is directly gated by calcium ions. J Biol Chem.

[CR34] Doihara H, Nozawa K, Kawabata-Shoda E, Kojima R, Yokoyama T, Ito H (2009). TRPA1 agonists delay gastric emptying in rats through serotonergic pathways. Naunyn Schmiedeberg’s Arch Pharmacol.

[CR35] Dolovich J, Back N, Arbesman CE (1970). Kinin-like activity in nasal secretions of allergic patients. Int Arch Allergy Appl Immunol.

[CR36] Eberhardt MJ, Filipovic MR, Leffler A, de la Roche J, Kistner K, Fischer MJ, Fleming T, Zimmermann K, Ivanovic-Burmazovic I, Nawroth PP, Bierhaus A, Reeh PW, Sauer SK (2012). Methylglyoxal activates nociceptors through transient receptor potential channel A1 (TRPA1): a possible mechanism of metabolic neuropathies. J Biol Chem.

[CR37] Eberhardt M, Dux M, Namer B, Miljkovic J, Cordasic N, Will C, Kichko TI, de la Roche J, Fischer M, Suarez SA, Bikiel D, Dorsch K, Leffler A, Babes A, Lampert A, Lennerz JK, Jacobi J, Marti MA, Doctorovich F, Hogestatt ED, Zygmunt PM, Ivanovic-Burmazovic I, Messlinger K, Reeh P, Filipovic MR (2014). H2S and NO cooperatively regulate vascular tone by activating a neuroendocrine HNO-TRPA1-CGRP signalling pathway. Nat Commun.

[CR38] Eid SR (2009) To feel or not to feel—targeting the heat sensor TRPV1 for pain treatment. In: The neurobiology of pain and analgesia. Keystone Symposium: Merck & Co., Inc., USA

[CR39] Eid SR, Crown ED, Moore EL, Liang HA, Choong KC, Dima S, Henze DA, Kane SA, Urban MO (2008). HC-030031, a TRPA1 selective antagonist, attenuates inflammatory- and neuropathy-induced mechanical hypersensitivity. Mol Pain.

[CR40] Engel MA, Leffler A, Niedermirtl F, Babes A, Zimmermann K, Filipovic MR, Izydorczyk I, Eberhardt M, Kichko TI, Mueller-Tribbensee SM, Khalil M, Siklosi N, Nau C, Ivanovic-Burmazovic I, Neuhuber WL, Becker C, Neurath MF, Reeh PW (2011). TRPA1 and substance P mediate colitis in mice. Gastroenterology.

[CR41] Engel MA, Khalil M, Mueller-Tribbensee SM, Becker C, Neuhuber WL, Neurath MF, Reeh PW (2012). The proximodistal aggravation of colitis depends on substance P released from TRPV1-expressing sensory neurons. J Gastroenterol.

[CR42] Garrison SR, Stucky CL (2014). Contribution of transient receptor potential ankyrin 1 to chronic pain in aged mice with complete Freund’s adjuvant-induced arthritis. Arthritis Rheum.

[CR43] Gracheva EO, Ingolia NT, Kelly YM, Cordero-Morales JF, Hollopeter G, Chesler AT, Sanchez EE, Perez JC, Weissman JS, Julius D (2010). Molecular basis of infrared detection by snakes. Nature.

[CR44] Gratzke C, Streng T, Waldkirch E, Sigl K, Stief C, Andersson KE, Hedlund P (2009). Transient receptor potential A1 (TRPA1) activity in the human urethra—evidence for a functional role for TRPA1 in the outflow region. Eur Urol.

[CR45] Gratzke C, Weinhold P, Reich O, Seitz M, Schlenker B, Stief CG, Andersson KE, Hedlund P (2010). Transient receptor potential A1 and cannabinoid receptor activity in human normal and hyperplastic prostate: relation to nerves and interstitial cells. Eur Urol.

[CR46] Hamada FN, Rosenzweig M, Kang K, Pulver SR, Ghezzi A, Jegla TJ, Garrity PA (2008). An internal thermal sensor controlling temperature preference in Drosophila. Nature.

[CR47] Hill K, Schaefer M (2007). TRPA1 is differentially modulated by the amphipathic molecules trinitrophenol and chlorpromazine. J Biol Chem.

[CR48] Hoffmann T, Kistner K, Miermeister F, Winkelmann R, Wittmann J, Fischer MJ, Weidner C, Reeh PW (2013). TRPA1 and TRPV1 are differentially involved in heat nociception of mice. Eur J Pain.

[CR49] Howard J, Bechstedt S (2004). Hypothesis: a helix of ankyrin repeats of the NOMPC-TRP ion channel is the gating spring of mechanoreceptors. Curr Biol: CB.

[CR50] Jang Y, Lee Y, Kim SM, Yang YD, Jung J, Oh U (2012). Quantitative analysis of TRP channel genes in mouse organs. Arch Pharm Res.

[CR51] Jaquemar D, Schenker T, Trueb B (1999). An ankyrin-like protein with transmembrane domains is specifically lost after oncogenic transformation of human fibroblasts. J Biol Chem.

[CR52] Jordt SE, Bautista DM, Chuang HH, McKemy DD, Zygmunt PM, Hogestatt ED, Meng ID, Julius D (2004). Mustard oils and cannabinoids excite sensory nerve fibres through the TRP channel ANKTM1. Nature.

[CR53] Kang K, Panzano VC, Chang EC, Ni L, Dainis AM, Jenkins AM, Regna K, Muskavitch MA, Garrity PA (2012). Modulation of TRPA1 thermal sensitivity enables sensory discrimination in Drosophila. Nature.

[CR54] Karashima Y, Talavera K, Everaerts W, Janssens A, Kwan KY, Vennekens R, Nilius B, Voets T (2009). TRPA1 acts as a cold sensor in vitro and in vivo. Proc Natl Acad Sci U S A.

[CR55] Kerstein PC, del Camino D, Moran MM, Stucky CL (2009). Pharmacological blockade of TRPA1 inhibits mechanical firing in nociceptors. Mol Pain.

[CR56] Kihara N, de la Fuente SG, Fujino K, Takahashi T, Pappas TN, Mantyh CR (2003). Vanilloid receptor-1 containing primary sensory neurones mediate dextran sulphate sodium induced colitis in rats. Gut.

[CR57] Kim HK, Park SK, Zhou JL, Taglialatela G, Chung K, Coggeshall RE, Chung JM (2004). Reactive oxygen species (ROS) play an important role in a rat model of neuropathic pain. Pain.

[CR58] Klionsky L, Tamir R, Gao B, Wang W, Immke DC, Nishimura N, Gavva NR (2007). Species-specific pharmacology of trichloro(sulfanyl)ethyl benzamides as transient receptor potential ankyrin 1 (TRPA1) antagonists. Mol Pain.

[CR59] Knowlton WM, Bifolck-Fisher A, Bautista DM, McKemy DD (2010). TRPM8, but not TRPA1, is required for neural and behavioral responses to acute noxious cold temperatures and cold-mimetics in vivo. Pain.

[CR60] Koivisto A, Hukkanen M, Saarnilehto M, Chapman H, Kuokkanen K, Wei H, Viisanen H, Akerman KE, Lindstedt K, Pertovaara A (2012). Inhibiting TRPA1 ion channel reduces loss of cutaneous nerve fiber function in diabetic animals: sustained activation of the TRPA1 channel contributes to the pathogenesis of peripheral diabetic neuropathy. Pharmacol Res: Off J Ital Pharmacol Soc.

[CR61] Kono T, Kaneko A, Omiya Y, Ohbuchi K, Ohno N, Yamamoto M (2013). Epithelial transient receptor potential ankyrin 1 (TRPA1)-dependent adrenomedullin upregulates blood flow in rat small intestine. Am J Physiology Gastrointest Liver Physiol.

[CR62] Kremeyer B, Lopera F, Cox JJ, Momin A, Rugiero F, Marsh S, Woods CG, Jones NG, Paterson KJ, Fricker FR, Villegas A, Acosta N, Pineda-Trujillo NG, Ramirez JD, Zea J, Burley MW, Bedoya G, Bennett DL, Wood JN, Ruiz-Linares A (2010). A gain-of-function mutation in TRPA1 causes familial episodic pain syndrome. Neuron.

[CR63] Kwan KY, Allchorne AJ, Vollrath MA, Christensen AP, Zhang DS, Woolf CJ, Corey DP (2006). TRPA1 contributes to cold, mechanical, and chemical nociception but is not essential for hair-cell transduction. Neuron.

[CR64] Kwan KY, Glazer JM, Corey DP, Rice FL, Stucky CL (2009). TRPA1 modulates mechanotransduction in cutaneous sensory neurons. J Neurosci.

[CR65] Kwon Y, Shim HS, Wang X, Montell C (2008). Control of thermotactic behavior via coupling of a TRP channel to a phospholipase C signaling cascade. Nat Neurosci.

[CR66] Lee KJ, Wang W, Padaki R, Bi V, Plewa CA, Gavva NR (2014). Mouse monoclonal antibodies to transient receptor potential ankyrin 1 act as antagonists of multiple modes of channel activation. J Pharmacol Exp Ther.

[CR67] Lehto SG, Youngblood BD, Zhang M, Weyer AD (2013) Evaluation of TRPA1 antagonists as pain therapeutics. Soc Neurosci Sci Prog 2013 Program#/Poster#: 740.713/OO711

[CR68] Lennertz RC, Kossyreva EA, Smith AK, Stucky CL (2012). TRPA1 mediates mechanical sensitization in nociceptors during inflammation. PLoS One.

[CR69] Liao M, Cao E, Julius D, Cheng Y (2013). Structure of the TRPV1 ion channel determined by electron cryo-microscopy. Nature.

[CR70] Lieu T, Jayaweera G, Zhao P, Poole DP, Jensen D, Grace M, McIntyre P, Bron R, Wilson YM, Krappitz M, Haerteis S, Korbmacher C, Steinhoff MS, Nassini R, Materazzi S, Geppetti P, Corvera CU, Bunnett NW (2014) The bile acid receptor TGR5 activates the TRPA1 channel to induce itch in mice. Gastroenterol 10.1053/j.gastro.2014.08.042 PMC4821165 25194674

[CR71] Liu B, Escalera J, Balakrishna S, Fan L, Caceres AI, Robinson E, Sui A, McKay MC, McAlexander MA, Herrick CA, Jordt SE (2013). TRPA1 controls inflammation and pruritogen responses in allergic contact dermatitis. FASEB J.

[CR72] Macpherson LJ, Geierstanger BH, Viswanath V, Bandell M, Eid SR, Hwang S, Patapoutian A (2005). The pungency of garlic: activation of TRPA1 and TRPV1 in response to allicin. Curr Biol.

[CR73] Materazzi S, Fusi C, Benemei S, Pedretti P, Patacchini R, Nilius B, Prenen J, Creminon C, Geppetti P, Nassini R (2012). TRPA1 and TRPV4 mediate paclitaxel-induced peripheral neuropathy in mice via a glutathione-sensitive mechanism. Pflugers Arch - Eur J Physiol.

[CR74] McGaraughty S, Chu KL, Perner RJ, Didomenico S, Kort ME, Kym PR (2010). TRPA1 modulation of spontaneous and mechanically evoked firing of spinal neurons in uninjured, osteoarthritic, and inflamed rats. Mol Pain.

[CR75] McNamara CR, Mandel-Brehm J, Bautista DM, Siemens J, Deranian KL, Zhao M, Hayward NJ, Chong JA, Julius D, Moran MM, Fanger CM (2007). TRPA1 mediates formalin-induced pain. Proc Natl Acad Sci U S A.

[CR76] Meseguer V, Alpizar YA, Luis E, Tajada S, Denlinger B, Fajardo O, Manenschijn JA, Fernandez-Pena C, Talavera A, Kichko T, Navia B, Sanchez A, Senaris R, Reeh P, Perez-Garcia MT, Lopez-Lopez JR, Voets T, Belmonte C, Talavera K, Viana F (2014). TRPA1 channels mediate acute neurogenic inflammation and pain produced by bacterial endotoxins. Nat Commun.

[CR77] Minett MS, Eijkelkamp N, Wood JN (2014). Significant determinants of mouse pain behaviour. PLoS One.

[CR78] Moparthi L, Survery S, Kreir M, Simonsen C, Kjellbom P, Hogestatt ED, Johanson U, Zygmunt PM (2014). Human TRPA1 is intrinsically cold- and chemosensitive with and without its N-terminal ankyrin repeat domain. Proc Natl Acad Sci U S A.

[CR79] Morquette B, Shi Q, Lavigne P, Ranger P, Fernandes JC, Benderdour M (2006). Production of lipid peroxidation products in osteoarthritic tissues: new evidence linking 4-hydroxynonenal to cartilage degradation. Arthritis Rheum.

[CR80] Motter AL, Ahern GP (2012). TRPA1 is a polyunsaturated fatty acid sensor in mammals. PLoS One.

[CR81] Mukhopadhyay I, Gomes P, Aranake S, Shetty M, Karnik P, Damle M, Kuruganti S, Thorat S, Khairatkar-Joshi N (2011). Expression of functional TRPA1 receptor on human lung fibroblast and epithelial cells. J Recept Signal Transduct Res.

[CR82] Munns C, AlQatari M, Koltzenburg M (2007). Many cold sensitive peripheral neurons of the mouse do not express TRPM8 or TRPA1. Cell Calcium.

[CR83] Nagata K, Duggan A, Kumar G, Garcia-Anoveros J (2005). Nociceptor and hair cell transducer properties of TRPA1, a channel for pain and hearing. J Neurosci.

[CR84] Nagatomo K, Ishii H, Yamamoto T, Nakajo K, Kubo Y (2010). The Met268Pro mutation of mouse TRPA1 changes the effect of caffeine from activation to suppression. Biophys J.

[CR85] Naik AK, Tandan SK, Dudhgaonkar SP, Jadhav SH, Kataria M, Prakash VR, Kumar D (2006). Role of oxidative stress in pathophysiology of peripheral neuropathy and modulation by N-acetyl-L-cysteine in rats. Eur J Pain.

[CR86] Nakayama K, Nakayama M, Iwabuchi M, Terawaki H, Sato T, Kohno M, Ito S (2008). Plasma alpha-oxoaldehyde levels in diabetic and nondiabetic chronic kidney disease patients. Am J Nephrol.

[CR87] Namer B, Seifert F, Handwerker HO, Maihofner C (2005). TRPA1 and TRPM8 activation in humans: effects of cinnamaldehyde and menthol. Neuroreport.

[CR88] Nassini R, Pedretti P, Moretto N, Fusi C, Carnini C, Facchinetti F, Viscomi AR, Pisano AR, Stokesberry S, Brunmark C, Svitacheva N, McGarvey L, Patacchini R, Damholt AB, Geppetti P, Materazzi S (2012). Transient receptor potential ankyrin 1 channel localized to non-neuronal airway cells promotes non-neurogenic inflammation. PLoS One.

[CR89] Niforatos W, Zhang XF, Lake MR, Walter KA, Neelands T, Holzman TF, Scott VE, Faltynek CR, Moreland RB, Chen J (2007). Activation of TRPA1 channels by the fatty acid amide hydrolase inhibitor 3′-carbamoylbiphenyl-3-yl cyclohexylcarbamate (URB597). Mol Pharmacol.

[CR90] Nozawa K, Kawabata-Shoda E, Doihara H, Kojima R, Okada H, Mochizuki S, Sano Y, Inamura K, Matsushime H, Koizumi T, Yokoyama T, Ito H (2009). TRPA1 regulates gastrointestinal motility through serotonin release from enterochromaffin cells. Proc Natl Acad Sci U S A.

[CR91] Obata K, Katsura H, Mizushima T, Yamanaka H, Kobayashi K, Dai Y, Fukuoka T, Tokunaga A, Tominaga M, Noguchi K (2005). TRPA1 induced in sensory neurons contributes to cold hyperalgesia after inflammation and nerve injury. J Clin Invest.

[CR92] Oh MH, Oh SY, Lu J, Lou H, Myers AC, Zhu Z, Zheng T (2013). TRPA1-dependent pruritus in IL-13-induced chronic atopic dermatitis. J Immunol.

[CR93] Petrus M, Peier AM, Bandell M, Hwang SW, Huynh T, Olney N, Jegla T, Patapoutian A (2007). A role of TRPA1 in mechanical hyperalgesia is revealed by pharmacological inhibition. Mol Pain.

[CR94] Poole DP, Pelayo JC, Cattaruzza F, Kuo YM, Gai G, Chiu JV, Bron R, Furness JB, Grady EF, Bunnett NW (2011) Transient receptor potential ankyrin 1 is expressed by inhibitory motoneurons of the mouse intestine. Gastroenterol 141:565–575, 575 e561–564 10.1053/j.gastro.2011.04.049 21689654

[CR95] Qian X, Francis M, Solodushko V, Earley S, Taylor MS (2013). Recruitment of dynamic endothelial Ca2+ signals by the TRPA1 channel activator AITC in rat cerebral arteries. Microcirculation.

[CR96] Rech JC, Eckert WA, Maher MP, Banke T, Bhattacharya A, Wickenden AD (2010). Recent advances in the biology and medicinal chemistry of TRPA1. Future Med Chem.

[CR97] Rooney L, Vidal A, D’Souza AM, Devereux N, Masick B, Boissel V, West R, Head V, Stringer R, Lao J, Petrus MJ, Patapoutian A, Nash M, Stoakley N, Panesar M, Verkuyl JM, Schumacher AM, Petrassi HM, Tully DC (2014). Discovery, optimization, and biological evaluation of 5-(2-(trifluoromethyl)phenyl)indazoles as a novel class of transient receptor potential A1 (TRPA1) antagonists. J Med Chem.

[CR98] Sawada Y, Hosokawa H, Hori A, Matsumura K, Kobayashi S (2007). Cold sensitivity of recombinant TRPA1 channels. Brain Res.

[CR99] Schwartz ES, Christianson JA, Chen X, La JH, Davis BM, Albers KM, Gebhart GF (2011). Synergistic role of TRPV1 and TRPA1 in pancreatic pain and inflammation. Gastroenterology.

[CR100] Schwartz ES, La JH, Scheff NN, Davis BM, Albers KM, Gebhart GF (2013). TRPV1 and TRPA1 antagonists prevent the transition of acute to chronic inflammation and pain in chronic pancreatitis. J Neurosci.

[CR101] Shields SD, Cavanaugh DJ, Lee H, Anderson DJ, Basbaum AI (2010). Pain behavior in the formalin test persists after ablation of the great majority of C-fiber nociceptors. Pain.

[CR102] Shigetomi E, Tong X, Kwan KY, Corey DP, Khakh BS (2012). TRPA1 channels regulate astrocyte resting calcium and inhibitory synapse efficacy through GAT-3. Nat Neurosci.

[CR103] Sisignano M, Park CK, Angioni C, Zhang DD, von Hehn C, Cobos EJ, Ghasemlou N, Xu ZZ, Kumaran V, Lu R, Grant A, Fischer MJ, Schmidtko A, Reeh P, Ji RR, Woolf CJ, Geisslinger G, Scholich K, Brenneis C (2012). 5,6-EET is released upon neuronal activity and induces mechanical pain hypersensitivity via TRPA1 on central afferent terminals. J Neurosci Off J Soc Neurosci.

[CR104] Smith MP, Beacham D, Ensor E, Koltzenburg M (2004). Cold-sensitive, menthol-insensitive neurons in the murine sympathetic nervous system. Neuroreport.

[CR105] Sotomayor M, Corey DP, Schulten K (2005). In search of the hair-cell gating spring elastic properties of ankyrin and cadherin repeats. Structure.

[CR106] Story GM, Peier AM, Reeve AJ, Eid SR, Mosbacher J, Hricik TR, Earley TJ, Hergarden AC, Andersson DA, Hwang SW, McIntyre P, Jegla T, Bevan S, Patapoutian A (2003). ANKTM1, a TRP-like channel expressed in nociceptive neurons, is activated by cold temperatures. Cell.

[CR107] Streng T, Axelsson HE, Hedlund P, Andersson DA, Jordt SE, Bevan S, Andersson KE, Hogestatt ED, Zygmunt PM (2008). Distribution and function of the hydrogen sulfide-sensitive TRPA1 ion channel in rat urinary bladder. Eur Urol.

[CR108] Sura L, Zima V, Marsakova L, Hynkova A, Barvik I, Vlachova V (2012). C-terminal acidic cluster is involved in Ca2+-induced regulation of human transient receptor potential ankyrin 1 channel. J Biol Chem.

[CR109] Takahashi N, Mizuno Y, Kozai D, Yamamoto S, Kiyonaka S, Shibata T, Uchida K, Mori Y (2008). Molecular characterization of TRPA1 channel activation by cysteine-reactive inflammatory mediators. Channels.

[CR110] Taylor-Clark TE, McAlexander MA, Nassenstein C, Sheardown SA, Wilson S, Thornton J, Carr MJ, Undem BJ (2008). Relative contributions of TRPA1 and TRPV1 channels in the activation of vagal bronchopulmonary C-fibres by the endogenous autacoid 4-oxononenal. J Physiol.

[CR111] Taylor-Clark TE, Undem BJ, Macglashan DW, Ghatta S, Carr MJ, McAlexander MA (2008). Prostaglandin-induced activation of nociceptive neurons via direct interaction with transient receptor potential A1 (TRPA1). Mol Pharmacol.

[CR112] Taylor-Clark TE, Nassenstein C, McAlexander MA, Undem BJ (2009). TRPA1: a potential target for anti-tussive therapy. Pulm Pharmacol Ther.

[CR113] Trevisan G, Materazzi S, Fusi C, Altomare A, Aldini G, Lodovici M, Patacchini R, Geppetti P, Nassini R (2013). Novel therapeutic strategy to prevent chemotherapy-induced persistent sensory neuropathy by TRPA1 blockade. Cancer Res.

[CR114] Trevisan G, Rossato MF, Hoffmeister C, Oliveira SM, Silva CR, Matheus FC, Mello GC, Antunes E, Prediger RD, Ferreira J (2013). Mechanisms involved in abdominal nociception induced by either TRPV1 or TRPA1 stimulation of rat peritoneum. Eur J Pharmacol.

[CR115] Trevisan G, Hoffmeister C, Rossato MF, Oliveira SM, Silva MA, Silva CR, Fusi C, Tonello R, Minocci D, Guerra GP, Materazzi S, Nassini R, Geppetti P, Ferreira J (2014). TRPA1 receptor stimulation by hydrogen peroxide is critical to trigger hyperalgesia and inflammation in a model of acute gout. Free Radic Biol Med.

[CR116] Trevisan G, Rossato MF, Tonello R, Hoffmeister C, Klafke JZ, Rosa F, Pinheiro KV, Pinheiro FV, Boligon AA, Athayde ML, Ferreira J (2014). Gallic acid functions as a TRPA1 antagonist with relevant antinociceptive and antiedematogenic effects in mice. Naunyn Schmiedeberg’s Arch Pharmacol.

[CR117] Tsutsumi M, Denda S, Ikeyama K, Goto M, Denda M (2010). Exposure to low temperature induces elevation of intracellular calcium in cultured human keratinocytes. J Invest Dermatol.

[CR118] Vilceanu D, Stucky CL (2010). TRPA1 mediates mechanical currents in the plasma membrane of mouse sensory neurons. PLoS One.

[CR119] Viswanath V, Story GM, Peier AM, Petrus MJ, Lee VM, Hwang SW, Patapoutian A, Jegla T (2003). Opposite thermosensor in fruitfly and mouse. Nature.

[CR120] Wang S, Dai Y, Fukuoka T, Yamanaka H, Kobayashi K, Obata K, Cui X, Tominaga M, Noguchi K (2008). Phospholipase C and protein kinase A mediate bradykinin sensitization of TRPA1: a molecular mechanism of inflammatory pain. Brain: J Neurol.

[CR121] Wang YY, Chang RB, Waters HN, McKemy DD, Liman ER (2008). The nociceptor ion channel TRPA1 is potentiated and inactivated by permeating calcium ions. J Biol Chem.

[CR122] Wei H, Hamalainen MM, Saarnilehto M, Koivisto A, Pertovaara A (2009). Attenuation of mechanical hypersensitivity by an antagonist of the TRPA1 ion channel in diabetic animals. Anesthesiology.

[CR123] Wei H, Chapman H, Saarnilehto M, Kuokkanen K, Koivisto A, Pertovaara A (2010). Roles of cutaneous versus spinal TRPA1 channels in mechanical hypersensitivity in the diabetic or mustard oil-treated non-diabetic rat. Neuropharmacology.

[CR124] Wei H, Koivisto A, Saarnilehto M, Chapman H, Kuokkanen K, Hao B, Huang JL, Wang YX, Pertovaara A (2011). Spinal transient receptor potential ankyrin 1 channel contributes to central pain hypersensitivity in various pathophysiological conditions in the rat. Pain.

[CR125] Wilson SR, Gerhold KA, Bifolck-Fisher A, Liu Q, Patel KN, Dong X, Bautista DM (2011). TRPA1 is required for histamine-independent, Mas-related G protein-coupled receptor-mediated itch. Nat Neurosci.

[CR126] Wilson SR, Nelson AM, Batia L, Morita T, Estandian D, Owens DM, Lumpkin EA, Bautista DM (2013). The ion channel TRPA1 is required for chronic itch. J Neurosci Off J Soc Neurosci.

[CR127] Wilson SR, The L, Batia LM, Beattie K, Katibah GE, McClain SP, Pellegrino M, Estandian DM, Bautista DM (2013). The epithelial cell-derived atopic dermatitis cytokine TSLP activates neurons to induce itch. Cell.

[CR128] Winterbourn CC, Kettle AJ (2000). Biomarkers of myeloperoxidase-derived hypochlorous acid. Free Radic Biol Med.

[CR129] Xiao B, Dubin AE, Bursulaya B, Viswanath V, Jegla TJ, Patapoutian A (2008). Identification of transmembrane domain 5 as a critical molecular determinant of menthol sensitivity in mammalian TRPA1 channels. J Neurosci.

[CR130] Zhang XF, Chen J, Faltynek CR, Moreland RB, Neelands TR (2008). Transient receptor potential A1 mediates an osmotically activated ion channel. Eur J Neurosci.

[CR131] Zurborg S, Yurgionas B, Jira JA, Caspani O, Heppenstall PA (2007). Direct activation of the ion channel TRPA1 by Ca2+. Nat Neurosci.

